# Strategies to Promote Healthy Eating Among University Students: A Qualitative Study Using the Nominal Group Technique

**DOI:** 10.3389/fnut.2022.821016

**Published:** 2022-02-02

**Authors:** Rungsaran Wongprawmas, Giovanni Sogari, Davide Menozzi, Cristina Mora

**Affiliations:** Department of Food and Drug, University of Parma, Parma, Italy

**Keywords:** nominal group technique (NGT), focus group, university students, canteen, healthy food, diet, young adult, Italy

## Abstract

**Introduction:**

The years spent at university are critical in terms of altering people's dietary patterns. This study aimed to: (1) understand the main dietary changes that students experience after starting university; (2) determine the personal and objective factors that hinder healthy eating, and (3) define possible strategies to facilitate healthier diets among university students.

**Methods:**

The nominal group technique (NGT) was used to elicit ideas from 39 students from the University of Parma, Italy. The sample comprised 16 freshmen and 23 non-freshmen. Participants prioritized and weighed their top five ideas regarding dietary changes, barriers to healthy eating, and possible strategies to maintain a healthy diet. A thematic analysis was conducted to compare the priorities across groups.

**Results:**

Forty-three themes were elected as the most significant changes related to diet, 39 themes related to personal barriers, 43 themes related to objective barriers, and 55 themes related to strategies. A lack of time for cooking, low financial availability, consumption of unvaried food or junk food, and gaining knowledge about food were identified as the main changes. Personal barriers to eating healthy were intrinsic (i.e., lack of willpower, personal gluttony, and little effort in cooking preparation), poor dietary information, and a busy lifestyle. Market and financial factors (i.e., the high price of healthy products and low financial availability), as well as social factors (i.e., the negative influence of social networks, childhood food education, and origin/tradition), emerged as objective barriers. Possible strategies that could encourage students to adopt a healthy diet include varying the food products offered in university canteens, including organizing spaces where students who prepare meals from home can warm up and eat their food. Student discounts at supermarkets and information on nutrition and a healthy diet were also identified as important ways of supporting students.

**Conclusion and Implication for Practice:**

In order to make students part of the solution, the NGT provided them with the opportunity to equally contribute their ideas and opinions about having a healthy diet in a university context. This could potentially lead to tailor-made solutions for policymakers, educators, and foodservice providers in promoting healthy eating habits.

## Introduction

For many young adults, leaving the parental home to go to university is a critical transition period, which includes new challenges such as taking charge of their own eating habits (1–3). During this time, several personal (intrinsic factors such as will, taste, and perception) and objective (extrinsic factors such as the environment or culture) factors (4) may discourage university students from following nutritional advice. As a result, many young people can go from being a normal weight to being overweight, which puts them at high risk for chronic disease (2, 3, 5).

Efforts to eat healthy foods are often overcome by certain perceived or encountered barriers, such as culinary traditions, social pressure, habits, and a lack of availability or the high price of healthy foods (3, 6, 7). In addition, a lack of knowledge or information, as well as a general lack of interest in making a change to one's diet may also prevent people from having a healthy diet (8). However, providing technical and detailed nutritional information is not always a successful strategy (9, 10). The reason is that healthy-eating guidelines have primarily been derived on an epidemiological basis (8), without considering consumers' personal and objective barriers.

The Health Belief Model (HBM) was developed in 1974 by Rosenstock (11). The HBM is an expectancy-value model that has been successfully used to predict healthy behaviors (12). Among the several factors included, the HBM postulates that individuals will engage in healthy behaviors when they perceive themselves as susceptible (e.g., eating changes occurring as a university student) and believe that the benefits of healthy behaviors exceed the costs (i.e., the perceived barriers). As a result, individuals are more likely to take preventive actions, such as maintaining a healthy diet (13). The HBM considers that cues to action (i.e., tools and strategies) can modify behavior-influencing variables by prompting actions.

In the past, several qualitative studies (some of which used focus groups) have been conducted on healthy eating perceptions and practices among young adults (3, 14–16). However, there is a lack of structural design to provide concrete and feasible implications based on the targeted group. To address this issue, the use of a nominal group technique (NGT) provides tailored suggestions that could be applied in a particular context.

NGT is a structured multi-step group technique that is used to generate and prioritize consensus responses to a carefully articulated question, which is intended to address specific issues (17–20). This technique has been used in a number of different contexts to generate ideas or allow a group to reach consensus (6, 20–22). The advantage of NGT over a traditional focus group is that it encourages equal contributions from everyone and facilitates discussion and prioritization, which leads participants to come to an agreement on an issue (1, 19, 23, 24). Therefore, the issues that are deemed important could be addressed first.

The purpose of this study is to better understand the factors that impede healthy eating behaviors among young adults. We utilized the NGT to elicit information from a group of Italian students and determine what changes occur in eating behavior after starting university, as well as the barriers associated with embracing a healthy diet. During the analysis, the sample was grouped by gender and year of enrollment (freshmen and non-freshmen) to provide variety and an in-depth interpretation of the results. The policy and managerial implications for campus settings are then provided and discussed.

## Materials and Methods

In our study, the NGT was applied to generate and prioritize participants' ideas about the perceived barriers of adopting healthy eating habits, as well as possible solutions. The NGT is a qualitative method of data collection that may be considered a highly structured focus group, which can guide face-to-face meetings (25). The NGT format is useful in that it allows participants to disclose creative and meaningful responses to a carefully articulated question that is intended to address specific information needs, as in the case of studies on healthy behavior (1, 19), including healthy diet (6, 26). It also allows for priorities between different groups of participants to be compared (19, 27).

### Participants

The sample consisted of 39 Italian university students (both male and female) that were recruited using advertisements that were handed out in and around the campus of the University of Parma, Italy. Snowball sampling was used to recruit participants. To be eligible, participants had to be enrolled as undergraduate or graduate students at the university. Written consent forms were provided to and approved by all participants at the beginning of the study, as well as an information sheet, which established the ground rules around confidentiality, respect, and protecting participants' identities. The study was approved by the Institutional Review Board (IRB) of the Ethical Committee of the University of Parma (Protocol ID: 41959, 17th November 2017).

### Data Collection

Seven NGT sessions were held at the university campus between January and April 2018. Each group was comprised of 3–8 individuals. The criteria of a maximum of seven NGTs and a group size of 2–14 participants was recommended by McMillan (19). The participants were full-time students aged 19–26 years. Two NGTs were made up of only freshmen students, three NGTs were only non-freshmen students, and the remaining NGT groups were made up of a mixture. Four NGTs were mixed genders, two were made up of only females, and one was exclusively male. All the sessions were conducted in Italian language, and each NGT session lasted approximately 90 min. The sessions were all initially videotaped, and the discussions were later transcribed and quality checked.

The structured questionnaire contained four questions adapted from the HBM (11) and was developed by a group of consumer researchers with expertise in attitudinal research within the field of nutrition. Based on this framework we developed the following questions:

1. What personal barriers can impede university students from eating healthily?
2. What objective barriers can impede university students from eating healthily?
3. When attending university, what changes occur to one's eating habits?
4. What are some techniques, strategies, or tools that might motivate university students to maintain a healthy diet?

Three researchers (one moderator and two facilitators) were trained to organize and facilitate the sessions. All sessions followed the same structure and procedures as those suggested by McMillan et al. (19).

Each focus group began with the first step in the NGT model, which involves the moderator providing a brief explanation of the purpose of the study and each participant introducing themselves. Each participant was then provided a pen and a worksheet with a specific question at the top of each page (for example, what personal barriers can impede university students from eating healthily?), which they worked on individually for 5 minto generate written responses. This second step of the protocol is the so-called silent generation of ideas during which time participants are not allowed to consult with each other or discuss their ideas (19, 28). After this step, we asked each participant to share their answers with the rest of the group using a “Round-robin” technique. While the responses were being shared, they were also verbatim recorded by the facilitators on a flip-chart, using the exact words spoken by the participants. Once all the responses were listed, the group discussion and clarification steps of the process began. The moderator discussed the responses to ensure that participants understood, thus enabling them to make informed decisions in the next step of selecting and ranking. In addition, through consensus, similar responses were combined with agreement from all participants (e.g., price, high price, high cost of healthy food products were combined as “high price of healthy products”). During the discussion process, participants could come up with new ideas that would then be added to the list in the flipchart.

The final step in the protocol is to select the top five themes from the generated list (flipchart) and prioritize (rank) the recorded ideas. Based on previous literature (19), we asked participants to rank the top five items that were most relevant to them (i.e., where one was the most salient idea and five was the least salient idea). It is important to note that even though participants' identities were not anonymous during the group discussion stage, the responses collected in the worksheet on the individual ranking were confidential.

All the steps of the NGT process were repeated for each question.

### Data Analysis

The data analysis of NGT combines both qualitative and quantitative methods. It involved coding the material of each session and constructing conceptual categories from the emerging codes, including calculating scores and prioritizing themes. Furthermore, individual comments from the transcription were cross-checked against the response sheets (i.e., the written comments) and information on the flipcharts (as recorded by the facilitator). As a result, this approach improved the clarity and depth of the results.

All the responses (themes) generated by each group were entered into Excel spreadsheets, along with the ranking of the responses. An initial review of the raw data in the spreadsheets (i.e., the original participant data) were used to identify any anomalies or nuances within the data. Then, the ranking was converted into scores, ranging from the least salient idea (1) to the most salient idea (5).

Responses from the seven NGT sessions that addressed the same questions were aggregated. Specifically, very similar themes were combined to create a new list of distinctive (non-overlapping) themes. Two authors (RW & GS) reviewed and discussed these aggregations to determine the final themes. The total score for each distinctive theme was computed by summing up the scores received from each participant and were combined across groups as the “sum of scores.” The relative importance of each theme was calculated as the proportion (%) of the total score for the theme on the maximum possible score (Participant number x 15). The priority list (“ranked priority”) was determined by considering the relative importance of the theme. If themes had equal relative importance, the voting frequency was then used to decide the ranking priority, whereby the higher the frequency of voting, the higher the priority.

The tables presented in the results section show the themes that were ranked in the top 10 for each question. A complete list of the responses is available in Supplementary File 1.

## Results

The demographic characteristics of the overall sample are shown in (Table 1). Thirty-nine university students (19–26 years old) participated in seven NGT sessions. The majority of the participants were female (56%) and had a normal weight (71%). The average age was 22 ± 2.2 years old with a mean body mass index (BMI) of 22.8 ± 3.2. Fifty-four percent of the participants were graduate students, and the rest were undergraduate students, of which freshmen made up the majority (89% of undergraduate students). Most participants (69%) lived with friends or flat mates.

**Table 1:** Participants' characteristics (*n* = 39).

**Characteristics**	***n* (%)**
Female	22 (56%)
Age mean (SD)	22 (2.2)
BMI mean (SD)	22.8 (3.2)
**BMI categories (*****n*** **= 38)**	
Underweight (BMI <18.50)	2 (5%)
Normal weight (18.50 ≤ BMI ≤ 24.99)	27 (69%)
Overweight (25.00 ≤ BMI ≤ 29.99)	8 (20%)
Obese (BMI ≥ 30.00)	1 (3%)
No response	1 (3%)
**Study**	
Undergraduate	18 (46%)
Graduate	21 (54%)
**Freshmen**	16 (41%)
**Living condition**	
With parents	7 (18%)
With friend/flat mate	27 (69%)
With partner	3 (8%)
Alone	2 (5%)

### Most Common Changes Related to Diet

A total of 43 themes related to dietary changes when attending university were generated; further details can be found in Supplementary Table S1. The aggregated responses that were ranked highly across all seven NGTs are presented in Table 2. “Lack of time for cooking,” “low financial availability,” “not having a varied diet” and “consumption of junk food” emerged as the responses with the greatest number of votes and frequencies across the seven groups. “Gaining knowledge about food” (selection and preparation of food) was also identified as an important theme.

**Table 2:** Aggregated results across the 7 groups on the main dietary changes since attending university.

**Idea**	**Total (*****n*** **= 39)**				**Freshmen (*****n*** **= 16)**				**Non-freshmen (*****n*** **= 23)**				**Female (*****n*** **= 22)**				**Male (*****n*** **= 17)**			
	**SS**	**RI**	**Freq**.	**Rank**	**SS**	**RI**	**Freq**.	**Rank**	**SS**	**RI**	**Freq**.	**Rank**	**SS**	**RI**	**Freq**.	**Rank**	**SS**	**RI**	**Freq**.	**Rank**
Lack of time for cooking	50	8.55	15	**1**	17	7.08	6	**5**	33	9.57	9	**1**	39	11.82	11	**1**	11	4.31	4	**8**
Low financial availability	48	8.21	15	**2**	25	10.42	7	**1**	23	6.67	8	**3**	26	7.88	8	**2**	22	8.63	7	**2**
Not having a varied diet	40	6.84	16	**3**	18	7.50	5	**4**	22	6.38	11	**4**	17	5.15	9	**5**	23	9.02	7	**1**
Consumption of junk food	36	6.15	11	**4**	21	8.75	7	**2**	15	4.35	4	**9**	17	5.15	5	**6**	19	7.45	6	**4**
Gaining knowledge about food (e.g., choosing/preparing food)	28	4.79	8	**5**	11	4.58	3	**9**	17	4.93	5	**6**	7	2.12	2	**19**	21	8.24	6	**3**
Change of daily routine	27	4.62	7	**6**	0	0.00	0	**28**	27	7.83	7	**2**	11	3.33	3	**13**	16	6.27	4	**6**
Exclusion of many healthy foods	22	3.76	6	**7**	0	0.00	0	**28**	22	6.38	6	**5**	8	2.42	2	**15**	14	5.49	4	**7**
Consuming cheaper foods	21	3.59	9	**8**	6	2.50	2	**17**	15	4.35	7	**8**	18	5.45	7	**3**	3	1.18	2	**21**
Low availability of healthy foods	18	3.08	6	**9**	18	7.50	6	**3**	0	0.00	0	**32**	18	5.45	6	**4**	0	0.00	0	**31**
Irregular meal times	17	2.91	5	**10**	7	2.92	3	**14**	10	2.90	2	**15**	15	4.55	4	**9**	2	0.78	1	**27**
High consumption of coffee	17	2.91	5	**10**	13	5.42	4	**8**	4	1.16	1	**24**	17	5.15	5	**6**	0	0.00	0	**31**
Study-related stress	17	2.91	5	**10**	0	0.00	0	**28**	17	4.93	5	**6**	17	5.15	5	**6**	0	0.00	0	**31**
Imbalanced diet due to lack of time	17	2.91	4	**13**	14	5.83	3	**7**	3	0.87	1	**25**	0	0.00	0	**35**	17	6.67	4	**5**
Careless shopping	14	2.39	5	**14**	0	0.00	0	**28**	14	4.06	5	**10**	11	3.33	4	**12**	3	1.18	1	**22**
Unhealthy lunch box	14	2.39	4	**15**	14	5.83	4	**6**	0	0.00	0	**32**	14	4.24	4	**10**	0	0.00	0	**31**
Little desire to cook	13	2.22	5	**17**	0	0.00	0	**28**	13	3.77	5	**11**	3	0.91	1	**26**	10	3.92	4	**9**
Consumption of ready-to-eat foods	12	2.05	5	**20**	10	4.17	4	**10**	2	0.58	1	**28**	7	2.12	3	**18**	5	1.96	2	**17**
Less healthy food regime	10	1.71	3	**22**	0	0.00	0	**28**	10	2.90	3	**14**	1	0.30	1	**30**	9	3.53	2	**10**

*SS, sum of scores; RI, relative importance; Freq, frequency of voting; Rank, ranked priority. The bold value is to highlight the ranked priorities of the themes*.

Themes varied slightly by gender and depending on whether participants were freshmen or non-freshmen. The most important change for the total sample non-freshmen and female participants was “lack of time for cooking”; for freshmen, it was “low financial availability”; and for males it was “not having a varied diet.”

### Personal Barriers to Maintaining a Healthy Diet

Overall, 39 themes were generated (see Supplementary Table S2). Table 3 presents the aggregated categories of personal barriers to following a healthy diet across all seven NGTs. Key personal barriers to eating healthy food were “lack of willpower,” “personal gluttony,” “little effort in cooking and preparation,” “poor dietary information,” and “lack of time during the day.”

**Table 3:** Aggregated results across the 7 groups on personal barriers to maintaining a healthy diet.

**Idea**	**Total (*****n*** **= 39)**				**Freshmen (*****n*** **= 16)**				**Non-freshmen (*****n*** **= 23)**				**Female (*****n*** **= 22)**				**Male (*****n*** **= 17)**			
	**SS**	**RI**	**Freq**.	**Rank**	**SS**	**RI**	**Freq**.	**Rank**	**SS**	**RI**	**Freq**.	**Rank**	**SS**	**RI**	**Freq**.	**Rank**	**SS**	**RI**	**Freq**.	**Rank**
Lack of willpower	62	10.60	17	**1**	19	7.92	5	**3**	43	12.46	12	**1**	28	8.48	8	**2**	34	13.33	9	**1**
Personal gluttony	60	10.26	22	**2**	32	13.33	11	**1**	28	8.12	11	**3**	36	10.91	14	**1**	24	9.41	8	**3**
Poor dietary information	44	7.52	12	**3**	24	10.00	5	**2**	20	5.80	7	**6**	13	3.94	3	**10**	31	12.16	9	**2**
Little effort in cooking and preparation	40	6.84	11	**4**	5	2.08	2	**16**	35	10.14	9	**2**	28	8.48	7	**3**	12	4.71	4	**6**
Lack of time during the day	34	5.81	9	**5**	10	4.17	2	**9**	24	6.96	7	**4**	24	7.27	6	**4**	10	3.92	3	**9**
Low financial availability	32	5.47	13	**6**	12	5.00	6	**7**	20	5.80	7	**6**	18	5.45	7	**6**	14	5.49	6	**5**
Lack of time for cooking	32	5.47	11	**7**	15	6.25	4	**4**	17	4.93	7	**8**	23	6.97	8	**5**	9	3.53	3	**10**
Lack of physical activity	27	4.62	10	**8**	5	2.08	1	**19**	22	6.38	9	**5**	16	4.85	7	**7**	11	4.31	3	**8**
Hectic daily routine	21	3.59	8	**9**	9	3.75	3	**10**	12	3.48	5	**12**	4	1.21	2	**22**	17	6.67	6	**4**
Challenge of following a balanced and varied diet	16	2.74	4	**10**	0	0.00	0	**29**	16	4.64	4	**9**	11	3.33	3	**12**	5	1.96	1	**19**
Individual laziness	15	2.56	4	**11**	15	6.25	4	**4**	0	0.00	0	**29**	15	4.55	4	**8**	0	0.00	0	**27**
Junk food consumption	14	2.39	4	**12**	14	5.83	4	**6**	0	0.00	0	**29**	14	4.24	4	**9**	0	0.00	0	**27**
Lack of willpower/self-control to maintain a healthy diet and lifestyle	13	2.22	4	**13**	0	0.00	0	**29**	13	3.77	4	**10**	5	1.52	2	**19**	8	3.14	2	**13**
Poor knowledge of food	13	2.22	4	**13**	0	0.00	0	**29**	13	3.77	4	**10**	4	1.21	1	**24**	9	3.53	3	**10**
Lack of interest in a healthy diet	12	2.05	3	**15**	0	0.00	0	**29**	12	3.48	3	**13**	0	0.00	0	**32**	12	4.71	3	**7**
Lack of time to eat	11	1.88	4	**18**	11	4.58	4	**8**	0	0.00	0	**29**	3	0.91	1	**27**	8	3.14	3	**12**

*SS, sum of scores; RI, relative importance; Freq, frequency of voting; Rank, ranked priority. The bold value is to highlight the ranked priorities of the themes*.

The primarily personal barrier for the total sample, non-freshmen and male participants was a “lack of willpower,” whereas for freshmen and female participants it was “personal gluttony.”

### Objective Barriers to Maintaining a Healthy Diet

The total number of themes generated was 43. Table 4 outlines the aggregated categories of objective barriers to maintaining a healthy diet across all seven NGTs. Further can be found in Supplementary Table S3. “High price of healthy products,” “low financial availability,” “negative influence of social networks,” “poor childhood food education,” and “origin and tradition” emerged as key objective barriers to eating healthy food among the university students.

**Table 4:** Aggregated results across the 7 groups on objective barriers to maintaining a healthy diet.

**Idea**	**Total (*****n*** **= 39)**				**Freshmen (*****n*** **= 16)**				**Non-freshmen (*****n*** **= 23)**				**Female (*****n*** **= 22)**				**Male (*****n*** **= 17)**			
	**SS**	**RI**	**Freq**.	**Rank**	**SS**	**RI**	**Freq**.	**Rank**	**SS**	**RI**	**Freq**.	**Rank**	**SS**	**RI**	**Freq**.	**Rank**	**SS**	**RI**	**Freq**.	**Rank**
High price of healthy products	69	11.79	20	**1**	28	11.67	8	**2**	41	11.88	12	**1**	30	9.09	9	**2**	39	15.29	11	**1**
Low financial availability	49	8.38	13	**2**	12	5.00	3	**6**	37	10.72	10	**2**	33	10.00	8	**1**	16	6.27	5	**4**
Negative influence of social networks	43	7.35	16	**3**	31	12.92	11	**1**	12	3.48	5	**9**	23	6.97	9	**3**	20	7.84	7	**2**
Poor childhood food education	36	6.15	10	**4**	0	0.00	0	**27**	36	10.43	10	**3**	21	6.36	6	**5**	15	5.88	4	**5**
Origin and tradition	35	5.98	12	**5**	22	9.17	7	**4**	13	3.77	5	**7**	23	6.97	8	**4**	12	4.71	4	**7**
Lack of time for cooking	34	5.81	8	**6**	26	10.83	6	**3**	8	2.32	2	**18**	20	6.06	5	**6**	14	5.49	3	**6**
Little information on healthy diet	23	3.93	9	**7**	9	3.75	3	**9**	14	4.06	6	**6**	3	0.91	3	**25**	20	7.84	6	**3**
Bad influence from friends, partners, and other people	23	3.93	8	**8**	6	2.50	3	**13**	17	4.93	5	**4**	18	5.45	6	**7**	5	1.96	2	**16**
Unhealthy family habits	21	3.59	7	**9**	14	5.83	4	**5**	7	2.03	3	**19**	17	5.15	5	**8**	4	1.57	2	**20**
Availability of unhealthy food	20	3.42	7	**10**	8	3.33	3	**10**	12	3.48	4	**10**	15	4.55	**5**	9	5	1.96	2	**16**
Unhealthy traditional food and habits	19	3.25	8	**11**	9	3.75	4	**7**	10	2.90	4	**13**	12	3.64	4	**11**	7	2.75	4	**12**
Lack of interest in a healthy diet	16	2.74	5	**12**	0	0.00	0	**27**	16	4.64	5	**5**	9	2.73	2	**15**	7	2.75	3	**13**
Misleading advertisements	15	2.56	4	**13**	5	2.08	1	**15**	10	2.90	3	**14**	15	4.55	4	**10**	0	0.00	0	**31**
Lack of time to maintain a healthy diet	13	2.22	5	**14**	0	0.00	0	**27**	13	3.77	5	**7**	1	0.30	1	**29**	12	4.71	4	**7**
External stimuli	12	2.05	4	**15**	0	0.00	0	**27**	12	3.48	4	**10**	2	0.61	1	**26**	10	3.92	3	**10**
Lack of organization during the day	11	1.88	3	**17**	0	0.00	0	**27**	11	3.19	3	**12**	0	0.00	0	**34**	11	4.31	3	**9**
Seasonal and local habits	9	1.54	4	**20**	9	3.75	4	**7**	0	0.00	0	**34**	9	2.73	4	**13**	0	0.00	0	**31**

*SS, sum of scores; RI, relative importance; Freq, frequency of voting; Rank, ranked priority. The bold value is to highlight the ranked priorities of the themes*.

The main objective barrier for the total sample, non-freshmen and male was the “high price of healthy products”; for freshmen participants it was the “negative influence of social networks”; and for female participants it was “low financial availability.”

### Strategies to Maintain a Healthy Diet

A total of 55 themes were generated (see Supplementary Table S4). The aggregated responses that ranked highly and could encourage students to adopt a healthy diet across all seven NGTs are presented in Table 5. “Varying food products offered in university canteens,” “student discount at supermarkets,” “better organization of university canteen areas” (so that students who prepare meal from home can warm up their food and eat it there), and “dissemination of information about healthy diet through seminars or courses” were identified as top strategies. “Reduce prices of sport facilities” was also mentioned as a crucial strategy though it was not directly related to healthy eating. This reflects the fact that students consider sport to be an important part of a healthy lifestyle.

**Table 5:** Aggregated results across the 7 groups for strategies to maintain a healthy diet.

**Idea**	**Total (*****n*** **= 39)**				**Freshmen (*****n*** **= 16)**				**Non-freshmen (*****n*** **= 23)**				**Female (*****n*** **= 22)**				**Male (*****n*** **= 17)**			
	**SS**	**RI**	**Freq**.	**Rank**	**SS**	**RI**	**Freq**.	**Rank**	**SS**	**RI**	**Freq**.	**Rank**	**SS**	**RI**	**Freq**.	**Rank**	**SS**	**RI**	**Freq**.	**Rank**
Varying food products offered in university canteens	53	9.06	14	**1**	26	10.83	7	**1**	27	7.83	7	**2**	42	12.73	11	**1**	11	4.31	3	**9**
Student discounts in supermarkets	36	6.15	10	**2**	21	8.75	6	**2**	15	4.35	4	**7**	26	7.88	6	**2**	10	3.92	4	**10**
Better organization of university canteen areas	33	5.64	10	**3**	0	0.00	0	**37**	33	9.57	10	**1**	17	5.15	5	**5**	16	6.27	5	**2**
Reduce prices of sport facilities	28	4.79	9	**4**	11	4.58	5	**6**	17	4.93	4	**6**	14	4.24	5	**9**	14	5.49	4	**3**
Dissemination of information about healthy diets through seminars or courses	24	4.10	8	**5**	0	0.00	0	**37**	24	6.96	8	**3**	15	4.55	5	**7**	9	3.53	3	**11**
Better organization of the day	24	4.10	7	**6**	11	4.58	3	**7**	13	3.77	4	**10**	20	6.06	6	**3**	4	1.57	1	**24**
Limit consumption of junk food in university canteens	23	3.93	5	**7**	0	0.00	0	**37**	23	6.67	5	**4**	5	1.52	1	**21**	18	7.06	4	**1**
Distribute appropriate information about correct diet	18	3.08	10	**8**	0	0.00	0	**37**	18	5.22	10	**5**	14	4.24	7	**8**	4	1.57	3	**22**
Discounted fruits and vegetables for university students	18	3.08	5	**9**	18	7.50	5	**3**	0	0.00	0	**34**	18	5.45	5	**4**	0	0.00	0	**37**
Greater economic availability	16	2.74	4	**10**	16	6.67	4	**4**	0	0.00	0	**34**	16	4.85	4	**6**	0	0.00	0	**37**
Availability of a broad range of healthy products	15	2.56	4	**11**	0	0.00	0	**37**	15	4.35	4	**7**	12	3.64	3	**11**	3	1.18	1	**27**
Attention to food labels	14	2.39	4	**12**	10	4.17	3	**8**	4	1.16	1	**24**	2	0.61	1	**37**	12	4.71	3	**8**
Discounted meals in university canteens	13	2.22	5	**14**	0	0.00	0	**37**	13	3.77	5	**9**	4	1.21	2	**25**	9	3.53	3	**11**
Varying the consumption of healthy products	13	2.22	3	**15**	8	3.33	2	**11**	5	1.45	1	**22**	0	0.00	0	**42**	13	5.10	3	**4**
Reduce prices of healthy foods	13	2.22	3	**15**	4	1.67	1	**22**	9	2.61	2	**15**	0	0.00	0	**42**	13	5.10	3	**4**
Prepare meals the day before	12	2.05	5	**17**	4	1.67	2	**20**	8	2.32	3	**16**	12	3.64	5	**10**	0	0.00	0	**37**
Advertising a healthy diet	12	2.05	4	**18**	12	5.00	4	**5**	0	0.00	0	**34**	0	0.00	0	**42**	12	4.71	4	**6**
Good food education from parents	12	2.05	4	**18**	8	3.33	3	**9**	4	1.16	1	**24**	0	0.00	0	**42**	12	4.71	4	**6**
Water delivering services	8	1.37	3	**23**	8	3.33	3	**9**	0	0.00	0	**34**	8	2.42	3	**12**	0	0.00	0	**37**

*SS, sum of scores; RI, relative importance; Freq, frequency of voting; Rank, ranked priority. The bold value is to highlight the ranked priorities of the themes*.

The top strategy for the total sample, freshmen and female participants was “varying food products offered in university canteens,” whereas this strategy was not deemed to be important by male participants (it ranked 9th). For males, “limited consumption of junk food in university canteens” was more important, while “better organization of university canteen areas” was the most important for non-freshmen participants.

## Discussion

The aim of this qualitative study was to provide in-depth insight into university students' dietary changes, barriers, and possible strategies that could encourage them to embrace a healthy diet. The differences in participants' responses by year of enrollment (freshmen vs. non-freshmen) and gender were also explored.

The dominant changes in eating habits included a lack of time to cook, limited budget, and consumption of unvaried food or junk food. On the other hand, gaining knowledge about food selection and preparation has also been identified as an important change. The results are in line with previous studies (3, 5, 15, 16, 29) that suggested time constraints, budgets, and consumption of unhealthy food are crucial changes that young adults experience when they go to university. Note that for non-freshmen students, gaining knowledge and experience in food decision and preparation are positive changes that could potentially lead to self-efficacy when it comes to having a healthy lifestyle (30). Time constraint and unbalanced or unvaried diet are crucial changes that are relevant to all participants.

Many studies have cited time constraints (3, 15) as a barrier to healthy eating. The participants highlighted that their hectic schedule did not allow them to have time to cook and eat properly. Since university students tend to prioritize studying and being accepted as peers, they tend to spend more time and resources on these issues compared to things like healthy eating (14, 16). Hence, they tend to eat fast and ready-to-eat foods, which might be high in calories and not very nutritious. In addition, our sample of participants were young and is in their peak physical stage; therefore, the effect of bad eating habits might not be visible at present, but will emerge later in their lives (5). To cope with these issues, attitudes and perceptions of healthy eating should be established by family and schools before enrolling in university. Time management is also a crucial skill that students should acquire before starting university to organize priorities during the study period.

Before attending university, most of the participants lived with their parents or guardians and were not used to doing food shopping or cooking by themselves. As a result, freshmen students might not know the importance of food prices, purchasing decisions, and cooking preparation. Since “poor childhood food education” and “origin and tradition” were reported among the most important objective barriers, we believe that these two aspects should be handled before starting university. For example, families could play an important role in encouraging their children to be self-sufficient in terms of diet—e.g., knowledge of food ingredients, awareness of healthy eating habits, and ability to cook basic dishes. Additionally, middle or high schools could offer courses on healthy cooking (e.g., simple recipes) and budget organizations to teach students the relevant skills. Off-campus social groups (e.g., club, unions) can also contribute by sharing places where cheap fruit and vegetables can be bought and by sharing quick and easy recipes with each other (31).

Our findings align with the conclusions of previous studies (29, 32) that identified differences between freshmen and non-freshmen students; this study found that freshmen students had less cooking skills than non-freshmen. Hence, financial and food availability are the main issues for freshmen students. At the same time, these aspects are less prominent for non-freshmen students, which indicates that they were able to gain these skills over time. Comparing by gender, junk food and fast food consumption was more prominent among males (29, 33), although females reported eating cheaper food. Participants reported that healthy food options, such as fruits and vegetables, are available and accessible in university canteens, but also mentioned that they are not varied enough and sold at higher prices compared to other food options.

The leading personal or subjective barriers to healthy eating identified by the participants included personal and intrinsic barriers, a lack of dietary information, and time constraints. Among the intrinsic barriers, the lack of willpower to adopt healthy eating patterns was the most frequently mentioned response, which is well-aligned with previous studies (7, 34, 35). Furthermore, we found that women perceived personal gluttony as the primary personal barrier. This might be because females tend to be more stressed than males; hence, they are more likely to eat sweet foods as a coping strategy (29, 36). A lack of physical activity was the fifth key barrier for non-freshmen students, while this issue was less important for other participant groups. Other studies have highlighted possible synergies of integrated public interventions aimed at improving young adults' health behaviors, such as physical activity and healthy food consumption (37).

A nutrition knowledge deficit was also mentioned as a personal barrier (14). Although this ranked second for freshmen participants, it ranked sixth among non-freshmen participants. This could imply that nutrition information was acquired after years of studying, since non-freshmen participants had completed at least one course on nutrition within the curricula.

The key objective barriers to healthy eating were market and financial factors (high price of healthy products, low financial availability), social factors (negative influence of social networks), childhood food education, and origin and tradition. The perception that healthy products have high prices was the top barrier for participants, which is in accordance with the findings of previous studies (3, 31). In other college food environments, such as in the US, most freshmen students are required to live on campus and eat every meal in the canteen, which includes a planned meal and an all-you-can-eat buffet. Therefore, the prices of healthy or unhealthy options are not the main factors affecting food choices. However, for non-freshmen students in the US, who tend to live off-campus, prices do play a role (1). In Italy, there is no such difference among freshmen and non-freshmen students since living on campus is not as common; therefore, students tend to either cook or go to canteens/restaurants/bars for meals.

Social and peer networks were cited as both barriers to, and facilitators of, healthy eating in other studies (14, 16, 31, 38). The influence of social media (i.e., Instagram, Facebook) was perceived as having a negative effect on the participants in this study. This highlights that social effects, especially from peers (including friends, acquaintances, and partners) are important in real-life or virtual social platforms (e.g., Instagram followings, Facebook friends).

Furthermore, childhood food education and family food habits are crucial since participants who grow up in a family that prioritizes healthy eating make better food choices at university (3, 14, 16). These results also indicate that participants who grew up with unhealthy family habits or traditions are unlikely to change, even though they know that they are unhealthy.

Individual places of origin and food traditions were also discussed as objective barriers to a healthy diet. For example, some participants mentioned that they consume a high amount of food during holidays or festivities at home (e.g., Christmas and Easter holidays). As a result, the high intake of traditional foods (appetizers, first course, second course, side dish, dessert, etc.) could contribute to a high caloric intake. Some students reported that during these festivities or other family reunion occasions they tend to eat unhealthy food (e.g., fried food) due to tradition and habits.

While there are differences between personal (intrinsic) and objective (extrinsic) factors (barriers), the results show that participants themselves did not clearly distinguish between these two types of barriers. In particular, a lack of time for cooking and a lack of information were considered to be both personal and objective barriers. This may hinder healthy eating habits even further, if people believe these are not personal issues that can be self-managed.

As suggested in previous literature (5, 14, 16, 37), changing the food environment (university canteen) was cited by students as the most promising strategy to encourage healthy eating. The changes included offering a variety of healthy food products in the canteen, offering discounted fruits and vegetables, and organizing spaces in canteens for students who bring food from home to be able to warm and consume food there. Additionally, offering healthy products throughout the university (for example, distributing healthy snacks like nuts, pre-cut fruits, and vegetables instead of high-calorie ones in vending machines) was also mentioned, although it was not ranked in the top five strategies.

Another frequently mentioned strategy was the dissemination of information on healthy diets through seminars and courses, which links to the previously mentioned belief that a lack of knowledge and information is a significant personal barrier. This finding is in accordance with the findings of previous studies, which held that improving knowledge could enable university students to eat more healthily (3, 14, 16, 31, 35). Therefore, offering a health class or seminar at the university (either on-site and online) is one way in which nutrition education can be improved. Nevertheless, students differ in terms of their personal characteristics and levels of nutrition-related knowledge, and more research regarding tailoring classes to address diverse groups of students (e.g., freshmen vs. non-freshmen) is required.

To overcome budget constraints, student discounts at supermarkets and lower prices of healthy food have also been proposed (16). Reducing the prices of sports facilities was brought up as a highly-ranked strategy, as found in previous studies (1, 15). This highlights the fact that eating nutritious food and performing physical activities are inseparable in most people's concept of healthy living, which therefore implies relevant strategies for university governing bodies (37).

### Practical Implications

For policy makers, universities, and teachers, providing tailored courses and seminars on knowledge of nutrition, cooking skills, budget organization, and time management for students with different characteristics and backgrounds is crucial. Furthermore, social networks could be useful tools for promoting knowledge and information about healthy eating; for example, this can be used to create social support groups among students who are seeking a healthier lifestyle. However, knowledge and information alone are not sufficient if the various healthy food options are unavailable. There is also a need for interventions that can promote convenient and healthy food options at the university in order to help students overcome time constraints and the perception that healthy food is labor-intensive (14).

There are opportunities for the industry and university canteens to provide healthy ready-to-eat foods that target young people who do not have much time but want to have a healthy lifestyle. For managers of university canteens, offering various healthy dishes and products could improve students' eating habits.

### Limitations

The NGTs were conducted with a non-probabilistic, purposively selected sample in a specific context; hence, it cannot be generalized to university students in other contexts. Nevertheless, it could form a basis for future studies that seek to find strategies and interventions to mitigate barriers and encourage students to eat healthily.

### Future Research

Further research should use the themes identified in this study to create and evaluate tailored intervention programs to promote healthy eating in the university. Future research might consider using the health belief model or the ecological model of health to design questions in the NGT study. Larger and more representative samples of university students (e.g., students from different disciplines or diverse geographical locations and contexts) should be included in future studies.

## Conclusion

This study identified barriers and strategies to facilitate healthy eating among university students. The most significant barriers included intrinsic factors (i.e., lack of willpower, personal gluttony, and lack of knowledge and information), busy lifestyle (i.e., lack of time), market and financial factors (i.e., high price of healthy products, and low financial availability), and social factors (i.e., influence of social networks, childhood food education, and food origin and tradition). The results of this study identify potential areas of intervention, such as offering healthier and various options of food in university canteens and vending machines, reducing prices of healthy food and sports facilities, and offering courses and seminars about healthy diets to students. The protocol of the NGT as an information-generating tool provides an opportunity for participants to share and contribute their ideas about having a healthy diet in a university context, thereby allowing them to feel that they were part of the solution. This highlights the importance of listening to students' opinions and experiences before designing and implementing interventions.

## Data Availability Statement

The original contributions presented in the study are included in the article/Supplementary Material, further inquiries can be directed to the corresponding author.

## Ethics Statement

The studies involving human participants were reviewed and approved by the Ethical Committee of the University of Parma (Protocol ID: 41959). The participants provided their written informed consent to participate in this study.

## Author Contributions

GS wrote the grant proposal, assisted with data analysis, and ran the NGT sessions. CM managed funding acquisition. GS, DM, and RW contributed to the conception and design of the study. DM and CM recruited the participants and contributed to manuscript revision. GS and CM prepared data collection material. RW performed the statistical analysis. RW and GS wrote the first draft of the manuscript. All authors read and approved the submitted version.

## Funding

This study was part of a wider project called CONSUMEHealth. Using consumer science to improve healthy eating habits and had received funding from the European Union's Horizon 2020 Research and Innovation Programme under the Marie Sklodowska-Curie Grant Agreement No. 749514.

## Conflict of Interest

The authors declare that the research was conducted in the absence of any commercial or financial relationships that could be construed as a potential conflict of interest.

## Publisher's Note

All claims expressed in this article are solely those of the authors and do not necessarily represent those of their affiliated organizations, or those of the publisher, the editors and the reviewers. Any product that may be evaluated in this article, or claim that may be made by its manufacturer, is not guaranteed or endorsed by the publisher.
