# Supplementary material for: Strategies to Promote Healthy Eating Among University Students: A Qualitative Study Using the Nominal Group Technique
Source: Front Nutr. 2022 Feb 2;9:821016. doi: 10.3389/fnut.2022.821016 (PMC8847783; doi:10.3389/fnut.2022.821016)
Supplement: Supplementary file 1 [file Table_1.DOCX]

**Table S1** The main dietary changes since attending university

| **No.** | **Idea** | **Sum of scores** | **Frequency** | **No. of groups** | **Ranked** |
| --- | --- | --- | --- | --- | --- |
|  |  | **(across groups)** | **of voting** | **endorsed idea** | **priority** |
| 1 | Lack of time for cooking | 50 | 15 | 4 | 1 |
| 2 | Low financial availability | 48 | 15 | 4 | 2 |
| 3 | Not having a varied diet | 40 | 16 | 5 | 3 |
| 4 | Consumption of junk food | 36 | 11 | 3 | 4 |
| 5 | Gaining knowledge about food (e.g., choosing/preparing food) | 28 | 8 | 3 | 5 |
| 6 | Change of daily routine | 27 | 7 | 1 | 6 |
| 7 | Exclusion of many healthy foods | 22 | 6 | 1 | 7 |
| 8 | Consuming cheaper foods | 21 | 9 | 2 | 8 |
| 9 | Low availability of healthy foods | 18 | 6 | 1 | 9 |
| 10 | Irregular meal times | 17 | 5 | 2 | 10 |
| 11 | High consumption of coffee | 17 | 5 | 2 | 10 |
| 12 | Study-related stress | 17 | 5 | 1 | 10 |
| 13 | Imbalanced diet due to lack of time | 17 | 4 | 1 | 13 |
| 14 | Careless shopping | 14 | 5 | 1 | 14 |
| 15 | Unhealthy lunch box | 14 | 4 | 1 | 15 |
| 16 | Change in personal diet | 13 | 6 | 2 | 16 |
| 17 | Little desire to cook | 13 | 5 | 1 | 17 |
| 18 | Independent life | 13 | 4 | 2 | 18 |
| 19 | Skip the main meals | 13 | 4 | 2 | 18 |
| 20 | Consumption of ready-to-eat foods | 12 | 5 | 4 | 20 |
| 21 | Increased responsibility in the diets | 10 | 4 | 1 | 21 |
| 22 | Less healthy food regime | 10 | 3 | 2 | 22 |
| 23 | Little time to eat | 10 | 3 | 1 | 22 |
| 24 | Know other traditional dishes | 9 | 3 | 1 | 24 |
| 25 | Low cooking skills | 8 | 3 | 1 | 25 |
| 26 | Hectic daily routine | 8 | 2 | 1 | 26 |
| 27 | Healthy eating to be more mental alert | 8 | 2 | 1 | 26 |
| 28 | Eat more at home | 8 | 2 | 1 | 26 |
| 29 | Social relationships affect food | 7 | 2 | 1 | 29 |
| 30 | Attention to food shopping | 6 | 2 | 1 | 30 |
| 31 | More time for yourself | 5 | 1 | 1 | 31 |
| 32 | High consumption of alcohol | 4 | 3 | 2 | 32 |
| 33 | Lack of cooking tools | 4 | 3 | 1 | 32 |
| 34 | Eating out | 4 | 2 | 2 | 34 |
| 35 | Decrease fatty food intake | 4 | 1 | 1 | 35 |
| 36 | Improve spaces for eating in the university | 3 | 1 | 1 | 36 |
| 37 | Don't live with parents | 3 | 1 | 1 | 36 |
| 38 | Difficulty in food procurement | 2 | 2 | 1 | 38 |
| 39 | Low physical activity | 2 | 1 | 1 | 39 |
| 40 | Fast meals due to lack of time | 1 | 1 | 1 | 40 |
| 41 | Disordered eating | 1 | 1 | 1 | 40 |
| 42 | Drink little water | 1 | 1 | 1 | 40 |
| 43 | More organized schedules | 1 | 1 | 1 | 40 |

**Table S2** Personal barriers to maintaining a healthy diet

| **No.** | **Idea** | **Sum of scores** | **Frequency** | **No. of groups** | **Ranked** |
| --- | --- | --- | --- | --- | --- |
|  |  | **(across groups)** | **of voting** | **endorsed idea** | **priority** |
| 1 | Lack of willpower | 62 | 17 | 4 | 1 |
| 2 | Personal gluttony | 60 | 22 | 6 | 2 |
| 3 | Poor dietary information | 44 | 12 | 3 | 3 |
| 4 | Little effort in cooking and preparation | 40 | 11 | 3 | 4 |
| 5 | Lack of time during the day | 34 | 9 | 2 | 5 |
| 6 | Low financial availability | 32 | 13 | 5 | 6 |
| 7 | Lack of time for cooking | 32 | 11 | 3 | 7 |
| 8 | Lack of physical activity | 27 | 10 | 4 | 8 |
| 9 | Hectic daily routine | 21 | 8 | 4 | 9 |
| 10 | Challenge of following a balanced and varied diet | 16 | 4 | 1 | 10 |
| 11 | Individual laziness | 15 | 4 | 1 | 11 |
| 12 | Consumption of junk food | 14 | 4 | 1 | 12 |
| 13 | Lack of willpower/self-control to maintain a healthy diet and lifestyle | 13 | 4 | 2 | 13 |
| 14 | Poor knowledge of food | 13 | 4 | 2 | 13 |
| 15 | Long preparation times | 12 | 4 | 2 | 15 |
| 16 | Lack of organization between work/study | 12 | 3 | 2 | 15 |
| 17 | Lack of interest in a healthy diet | 12 | 3 | 2 | 15 |
| 18 | Lack of time to eat | 11 | 4 | 1 | 18 |
| 19 | Stress | 11 | 4 | 1 | 18 |
| 20 | Stress from external factors | 8 | 3 | 1 | 20 |
| 21 | Food intolerance | 8 | 2 | 2 | 21 |
| 22 | Little time spent on choosing and purchasing foods | 7 | 3 | 1 | 22 |
| 23 | Lack of initiative to adopt a healthy lifestyle | 7 | 3 | 1 | 22 |
| 24 | Psychological problems triggering eating disorders | 7 | 2 | 1 | 24 |
| 25 | Eat more than necessary because of eating out with friends | 6 | 4 | 1 | 25 |
| 26 | Poor food education since childhood | 6 | 3 | 2 | 26 |
| 27 | Work-related commitments | 6 | 2 | 1 | 27 |
| 28 | Food availability | 5 | 2 | 1 | 28 |
| 29 | Attention to seasonality of products | 5 | 2 | 1 | 28 |
| 30 | Personal tastes | 5 | 2 | 1 | 28 |
| 31 | Large consumption of ready-made high-fat foods | 5 | 2 | 1 | 28 |
| 32 | Unhealthy family habits | 4 | 2 | 1 | 32 |
| 33 | Irregular meal times | 4 | 1 | 1 | 33 |
| 34 | Negative influence of social networks | 3 | 3 | 2 | 34 |
| 35 | Unhealthy food traditions | 3 | 2 | 1 | 35 |
| 36 | Bad mood | 3 | 1 | 1 | 36 |
| 37 | Poor diet | 3 | 1 | 1 | 36 |
| 38 | Little creativeness in cooking | 3 | 1 | 1 | 36 |
| 39 | Excessive alcohol consumption | 2 | 1 | 1 | 39 |

**Table S3** Objective barriers to maintaining a healthy diet

| **No.** | **Idea** | **Sum of scores** | **Frequency** | **No. of groups** | **Ranked** |
| --- | --- | --- | --- | --- | --- |
|  |  | **(across groups)** | **of voting** | **endorsed idea** | **priority** |
| 1 | High price of healthy products | 69 | 20 | 6 | 1 |
| 2 | Low financial availability | 49 | 13 | 4 | 2 |
| 3 | Negative influence of social networks | 43 | 16 | 4 | 3 |
| 4 | Poor childhood food education | 36 | 10 | 3 | 4 |
| 5 | Origin and tradition | 35 | 12 | 4 | 5 |
| 6 | Lack of time for cooking | 34 | 8 | 3 | 6 |
| 7 | Little information on healthy diet | 23 | 9 | 3 | 7 |
| 8 | Bad influence from friends, partners, and other people | 23 | 8 | 3 | 8 |
| 9 | Unhealthy family habits | 21 | 7 | 3 | 9 |
| 10 | Availability of unhealthy food | 20 | 7 | 2 | 10 |
| 11 | Unhealthy traditional food and habits | 19 | 8 | 2 | 11 |
| 12 | Lack of interest in a healthy diet | 16 | 5 | 2 | 12 |
| 13 | Misleading advertisements | 15 | 4 | 1 | 13 |
| 14 | Lack of time to maintain a healthy diet | 13 | 5 | 2 | 14 |
| 15 | External stimuli | 12 | 4 | 1 | 15 |
| 16 | Dietary choices influenced by discounts in retail stores | 11 | 5 | 1 | 16 |
| 17 | Lack of organization during the day | 11 | 3 | 2 | 17 |
| 18 | Cultural background | 11 | 3 | 2 | 17 |
| 19 | Revenue-oriented food companies | 10 | 2 | 1 | 19 |
| 20 | Seasonal and local habits | 9 | 4 | 1 | 20 |
| 21 | Negative effect of hectic daily routine on meal quality | 9 | 3 | 1 | 21 |
| 22 | Personal gluttony | 9 | 2 | 1 | 22 |
| 23 | Stress from external factors | 7 | 3 | 1 | 23 |
| 24 | Lack of attention in food choices | 7 | 2 | 1 | 24 |
| 25 | Lack of time to eat | 6 | 4 | 1 | 25 |
| 26 | Choosing unhealthy foods | 6 | 2 | 1 | 26 |
| 27 | Lack of self-control to maintain a healthy lifestyle | 6 | 2 | 2 | 26 |
| 28 | Lack of information from authorities | 5 | 2 | 1 | 28 |
| 29 | Low intakes of nutrients | 5 | 1 | 1 | 29 |
| 30 | Lack of food culture | 5 | 1 | 1 | 29 |
| 31 | Lack of family support in following a diet | 4 | 3 | 1 | 31 |
| 32 | Expensive sport services | 4 | 1 | 1 | 32 |
| 33 | Large family size | 4 | 1 | 1 | 32 |
| 34 | Lack of encouragement | 4 | 1 | 1 | 32 |
| 35 | Follow new food trends | 4 | 1 | 1 | 32 |
| 36 | Unavailability of healthy foods | 3 | 2 | 1 | 36 |
| 37 | Junk food consumption | 3 | 1 | 1 | 37 |
| 38 | Eating out | 2 | 2 | 2 | 38 |
| 39 | Lack of time in the day | 2 | 2 | 1 | 38 |
| 40 | Do not follow a balanced diet | 2 | 1 | 1 | 40 |
| 41 | Little varied diet | 2 | 1 | 1 | 40 |
| 42 | Personal eating habits | 1 | 1 | 1 | 42 |
| 43 | Special occasions | 1 | 1 | 1 | 42 |

**Table S4** Strategies to maintain a healthy diet

| **No.** | **Idea** | **Sum of scores** | **Frequency** | **No. of groups** | **Ranked** |
| --- | --- | --- | --- | --- | --- |
|  |  | **(across groups)** | **of voting** | **endorsed idea** | **priority** |
| 1 | Varying food products offered in university canteens | 53 | 14 | 3 | 1 |
| 2 | Student discounts in supermarkets | 36 | 10 | 3 | 2 |
| 3 | Better organization of university canteen areas | 33 | 10 | 2 | 3 |
| 4 | Reduce prices of sport facilities | 28 | 9 | 3 | 4 |
| 5 | Dissemination of information about healthy diets through seminars or courses | 24 | 8 | 2 | 5 |
| 6 | Better organization of the day | 24 | 7 | 3 | 6 |
| 7 | Limit consumption of junk food in university canteens | 23 | 5 | 1 | 7 |
| 8 | Distribute appropriate information about correct diet | 18 | 10 | 2 | 8 |
| 9 | Discounted fruits and vegetables for university students | 18 | 5 | 1 | 9 |
| 10 | Greater economic availability | 16 | 4 | 2 | 10 |
| 11 | Availability of a broad range of healthy products | 15 | 4 | 1 | 11 |
| 12 | Attention to healthy diet | 14 | 5 | 2 | 12 |
| 13 | Attention to food labels | 14 | 4 | 1 | 12 |
| 14 | Discounted meals in university canteens | 13 | 5 | 1 | 14 |
| 15 | Varying the consumption of healthy products | 13 | 3 | 1 | 15 |
| 16 | Reduce prices of healthy foods | 13 | 3 | 2 | 15 |
| 17 | Prepare meals the day before | 12 | 5 | 1 | 17 |
| 18 | Advertising a healthy diet | 12 | 4 | 1 | 18 |
| 19 | Good food education from parents | 12 | 4 | 2 | 18 |
| 20 | Limit the purchasing from vending machines | 11 | 3 | 1 | 20 |
| 21 | Vending machines containing healthy foods,  dried fruits | 10 | 3 | 1 | 21 |
| 22 | Prepare meal at home | 10 | 3 | 1 | 21 |
| 23 | Positive friendship network | 8 | 3 | 1 | 23 |
| 24 | Water delivering services | 8 | 3 | 1 | 23 |
| 25 | Organization of courses related to diet with credits | 8 | 2 | 1 | 25 |
| 26 | Avoid lessons and/or exams during lunch breaks | 7 | 5 | 1 | 26 |
| 27 | Cooking familiarity | 7 | 3 | 1 | 27 |
| 28 | Eating meals with friends/family members | 7 | 2 | 1 | 28 |
| 29 | Get more information about sports activities | 7 | 2 | 1 | 28 |
| 30 | Self-motivation | 6 | 2 | 1 | 30 |
| 31 | Increase the use of social media / apps | 5 | 3 | 1 | 31 |
| 32 | Try new food recipes | 5 | 2 | 1 | 32 |
| 33 | Do physical activity | 5 | 2 | 2 | 32 |
| 34 | Lowering stress | 5 | 2 | 1 | 32 |
| 35 | Increase knowledge | 5 | 2 | 1 | 32 |
| 36 | Health awareness day | 5 | 2 | 1 | 32 |
| 37 | Controlled and justified food shopping | 5 | 1 | 1 | 37 |
| 38 | Consultations with a dietician | 5 | 1 | 1 | 37 |
| 39 | Planning daily meals | 4 | 2 | 1 | 39 |
| 40 | Dinners with healthy food | 4 | 2 | 1 | 39 |
| 41 | Follow new food studies | 4 | 2 | 1 | 39 |
| 42 | Spend little time away from home | 4 | 1 | 1 | 42 |

**Table S4** (Continue)

| **No.** | **Idea** | **Sum of scores** | **Frequency** | **No. of groups** | **Ranked** |
| --- | --- | --- | --- | --- | --- |
|  |  | **(across groups)** | **of voting** | **endorsed idea** | **priority** |
| 43 | Make a shopping list | 4 | 1 | 1 | 42 |
| 44 | Getting familiarity with kitchen tools | 3 | 3 | 1 | 44 |
| 45 | Research on internet or tv programs | 3 | 2 | 1 | 45 |
| 46 | Provide student meal vouchers | 3 | 2 | 1 | 45 |
| 47 | Pay attention to food prices | 3 | 1 | 1 | 47 |
| 48 | Social services supporting healthy diet | 3 | 1 | 1 | 47 |
| 49 | Good example from friends | 3 | 1 | 1 | 47 |
| 50 | App to remember to drink water daily | 3 | 1 | 1 | 47 |
| 51 | Diet awareness | 3 | 1 | 1 | 47 |
| 52 | Negative influence from social networks | 2 | 1 | 1 | 52 |
| 53 | Use post-it notes / cards to remind yourself to eat healthy foods | 2 | 1 | 1 | 52 |
| 54 | Improving city's bicycle service | 1 | 1 | 1 | 54 |
| 55 | Providing cooking recipes | 1 | 1 | 1 | 54 |
